# Spondylodiscitis and Its Mimickers: A Pictorial Review

**DOI:** 10.3390/biomedicines12112566

**Published:** 2024-11-09

**Authors:** Claudia Lucia Piccolo, Alberta Villanacci, Federica Di Stefano, Nicoletta Fusco, Davide Roberto Donno, Massimo Cristofaro, Fabrizio Taglietti, Stefania Ianniello

**Affiliations:** 1 Department of Radiology, Fondazione Policlinico Campus Bio-Medico, 00128 Rome, Italy; 2 Department of Radiology, National Institute for Infectious Disease “Lazzaro Spallanzani”, 00149 Rome, Italy; alberta.villanacci@inmi.it (A.V.); federica.distefano@inmi.it (F.D.S.); nicoletta.fusco@inmi.it (N.F.); massimo.cristofaro@inmi.it (M.C.); stefania.ianniello@inmi.it (S.I.); 3 Department of Infectious Disease, National Institute for Infectious Disease “Lazzaro Spallanzani”, 00149 Rome, Italy; davideroberto.donno@inmi.it (D.R.D.); fabrizio.taglietti@inmi.it (F.T.)

**Keywords:** vertebral infection, spondylodiscitis, tubercular infection, spine infection, paravertebral abscess

## Abstract

Spondylodiscitis is an infection of the intervertebral disc, the adjacent vertebral body, and/or contiguous structures due to the introduction of infectious agent, usually by the hematogenous route. Imaging is crucial in assessing bacterial and tubercular spondylodiscitis, as well as their associated complications. Magnetic resonance imaging in particular can clearly depict osteo-structural changes in the vertebral body and the associated disc, as well as any soft-tissue complications, such as paravertebral abscess and/or epidural abscess, improving disease characterization and helping to recognize the agent involved. Nevertheless, other non-infectious diseases may mimic imaging appearances of spondylodiscitis and one should be aware of these conditions when interpreting MR images, which include Modic type I degenerative changes, ankylosing spondylitis, acute Schmorl’s node, porotic fractures, and spinal neuropathy arthropathy. This pictorial review aims at describing imaging findings of bacterial and non-bacterial spondylodiscitis, complications, and those pathologies that mimic these infections.

## 1. Epidemiology

Spondylodiscitis is an infection of the intervertebral disc, the adjacent vertebral body, and/or contiguous structures due to the introduction of the infectious agent, usually by the hematogenous route. It can be due because of an infection in a distant site (endocarditis, abscess, urinary tract infection, lung or pelvic infection), following a surgical intervention in a distant site, or can complicate a local infection that becomes systemic or results from intravenous use of illicit drugs [1].

In adults, the site of origin is usually the endplate which is necrotized by a septic embolus, and the disc is involved later; in adults, the intervertebral disc is avascular and thus emboli cause ischemia and infarction of the vertebral endplates with subsequent bone destruction and disc involvement. In infants, the disease instead affects the disc first and only later the endplates, whose perfusion is granted by the anastomoses of the intraosseous arteries and their branches penetrating the disc.

The incidence of spondylodiscitis averages seven cases per million, with an annual increase of about 7%, with men being affected three times more often than women, and a bimodal distribution; in fact, it is diagnosed in the first and second decades of life in children, whereas in adults it tends to affect patients in the fifth and sixth decades of life. 

Specifically, pyogenic discitis is more common in older patients (59–69 years), with a male preponderance, whereas tubercular spondylodiscitis affects a broader age range with a bimodal peak in developed countries, where the first peak is composed of young immigrants or HIV-coinfected people, whereas the second peak comprises immunosuppressed or older patients with comorbidities. 

Tubercular discitis more commonly affects female patients.

Brucellar spondylodiscitis occurs in older patients, but is more homogeneously distributed between both sexes.

In addition to age, risk factors include diabetes mellitus, malnutrition and disorders inducing loss of weight, steroid therapy, HIV infection, immunosuppression, oncologic history, renal failure, rheumatic diseases, and spinal surgery. Specifically, a long duration of surgery, high blood loss, and the quantity of operations are significant risk factors, leading to a spinal infection rate of 1–4% after spinal surgery. Particular attention must be paid when dealing with surgically treated obese patients, as a subcutaneous fat thickness > 50 mm may lead to a significantly higher risk of postoperative infection and colonization by microorganisms [2,3,4,5,6,7].

## 2. Pathogenesis and Clinical Presentation

Spondylodiscitis refers to an infection that involves both the intervertebral disc and the adjacent vertebral bodies, leading to osteomyelitis in the regions near the disc. Accurate identification of the causative pathogen is essential for initiating appropriate treatment promptly and determining whether orthopedic intervention is necessary. Factors such as the patient’s age, overall health, immune status, and the specific infectious agent influence the clinical and radiological presentation, as well as the progression of the infection [8].

Spondylodiscitis may have bacterial or non-bacterial etiology (fungi and parasites), and, in the former case, a relatively broad spectrum of pathogenic agents is considered. The prevalence of tuberculosis and pyogenic causative organisms is common in developing nations, but in recent times the list of causative organisms has significantly expanded in scope, even including fungi [8].

The causes depend mainly on the location of the infection, as different pathogens exhibit different localizations.

From an etiological point of view, on the other hand, the infections can be pyogenic and granulomatous [4]. Pyogenic infections typically affect the anterior components of the spinal column, whereas involvement of the posterior arch, though uncommon, usually suggests a tubercular cause. The dura mater acts as a strong barrier against infection, making meningitis and myelitis rare complications in cases of spondylodiscitis [4].

The largest proportion of spondylodiscitis cases with epidural abscesses are caused by *Staphylococcus aureus*. *Escherichia coli* is often responsible for spondylitis/spondylodiscitis and paraspinal infections. Only 0.5% of cases are fungal in etiology, and viral and parasitic infections are rare. However, the vertebra is the most common site of fungal osteomyelitis, with about one-third of fungal infections of the spine being due to Aspergillus and another third to Candida. No causative organism is isolated in 21–34% of cases [9,10]. 

Pyogenic spondylodiscitis through hematogenous spread mostly affects the lumbar spine (58%) followed by the thoracic (30%) and cervical spine (11%). In cases of spondylodiscitis affecting the cervical region, muscle contraction, rigidity, and stiffness may occur. If the patient is experiencing dysphagia or difficulty breathing, the possibility of a retropharyngeal abscess should always be considered [3,4,5].

This differs from tuberculous (TB) disease, which mainly affects the thoracic spine and often more than two levels; this can be a distinctive feature of pyogenic infection [3].

Single-level involvement is seen in 65% of patients, while 35% have multiple-level involvement, including 10% with non-contiguous levels. The median delay to diagnosis for pyogenic infection is 30 days, with microbiological diagnosis established in approximately three-quarters of cases. The median duration of antibiotic therapy is 148 days. Although conservative treatment can be effective for 70% of cases of pyogenic infection, surgery may be required in over 50% of cases for all causes. For pyogenic infection, complete healing without disability occurs in over three-quarters of cases; there is an overall healing rate of 91%, with 24% experiencing residual disabilities and a mortality rate of 8%. Adverse prognostic factors include negative microbiological culture, neurological impairment at diagnosis, and endocarditis [6,7].

The Infectious Diseases Society of America guidelines recommend diagnosing spondylodiscitis based on clinical factors (such as back or neck pain, fever, leukocyte count, and inflammatory markers like C-reactive protein and procalcitonin), along with pathogen identification from blood cultures and intraoperative tissue samples and histopathological examination [5]. They also recognize a few elements useful to improve the diagnostic work-up in spondylodiscitis management, such as segmental instability, the presence of an abscess, neurological compression, and the focus of infection.

**Scheme 1:**
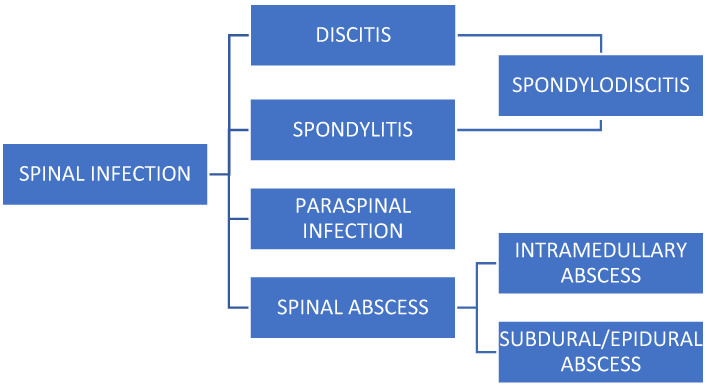
Classification of spinal infections based on the location of the infection.

The classification of spinal infection (SI) is usually based on the location of the infection, discriminating between intraspinal infection, bone infection, disc and bone infection, and soft tissue infection (such as muscle abscess) (Scheme 1).

The classification of spinal infections is designed to guide treatment choices, with the primary goals of preserving spinal stability, relieving pain, preventing or minimizing neurological deficits, and reducing the risk of recurrence. Pola et al. [11] have proposed a new clinical–radiological classification system to establish a standardized treatment algorithm. This system identifies three main categories based on primary criteria: bone destruction or segmental instability, epidural abscesses, and neurological impairment. Subcategories are further defined by secondary criteria. Cases without segmental instability or neurological impairment are treated conservatively, with surgery reserved for instances of significant bone loss or neurological deterioration. All patients were monitored with clinical and radiological follow-up for two years. This classification encompasses all orthopedic treatment options, helping surgeons to reduce complications, control costs, and prevent overtreatment.

The early detection of SI is still challenging because of the unspecific presentation; early diagnosis is hindered by the fact that the majority of patients do not show any signs of prior infection, and when present, they have a low specificity. Diagnosis should be supported by clinical, laboratory, and imaging findings. As mentioned before, clinical routine markers are suitable for diagnosis and also for evaluation of the treatment response. C-reactive protein (CRP) is elevated in more than 90% of acute spondylodiscitis cases and is a sensitive marker for bacterial infection; moreover, is considered the most specific marker for treatment response, as it returns to normal levels rapidly after successful treatment. Furthermore, CRP is elevated in more than 90% of acute spondylodiscitis cases and is a sensitive marker for bacterial infection. The erythrocyte sedimentation rate (ESR) is a sensitive biomarker for infection but has a low specificity too. It can be used as a marker for therapeutic response only in some specific cases, as ESR is still increased in 50% of patients with good clinical outcomes. The white blood cell (WBC) count, however, is less useful than ESR and CRP, as the presence of a normal WBC count does not exclude the diagnosis of spinal infection.

Procalcitonin (PCT) is less sensitive than CRP in patients with spinal infection, although its sensitivity increases by increasing the number of infected sites. Thus, patients with elevated PCT levels should be considered as suffering from combined infection. Blood and urine cultures should be collected when a spinal infection is suspected, before starting any antibiotic therapy. Up to 59% of positive blood cultures identify the etiological microorganism in patients with monomicrobial pyogenic spondylodiscitis [3].

Because biopsy can be superior to blood culture, computed tomography (CT)-guided biopsy should be considered to obtain the correct micro-organism etiology [3]. In tuberculosis (TB) disease, the erythrocyte sedimentation rate (ESR) is often elevated above 20 mm/h and decreases with treatment. C-reactive protein (CRP) is considered a more reliable marker of recent infection than ESR. Serological testing for IgM and IgG antibodies, which rise during the acute and chronic stages of TB infection, respectively, is not recommended because it cannot differentiate between the two stages or between spontaneous infection and BCG vaccination. While the Mantoux tuberculin skin test is endorsed by the World Health Organization (WHO) for use in low-income countries, its diagnostic value is limited in endemic areas and it may yield false negatives in immunocompromised individuals. Interferon-gamma (IFN-γ) release assays, which measure the IFN-γ produced in response to Mycobacterium tuberculosis antigens, and whole blood enzyme-linked immunosorbent assays (ELISAs) are also used in diagnosing latent TB. These tests are specific and can distinguish latent from active TB, but they cannot differentiate between natural TB infection and BCG vaccination. The Gene Xpert MTB/RIF test is a rapid, fully automated diagnostic tool that can provide results in under 90 min. Spinal TB diagnosis is based on a combination of clinical symptoms, characteristic MRI findings, and confirmation through culture and sensitivity testing, Gene Xpert PCR, or histological analysis.

## 3. Diagnostic Work-Up

### 3.1. Conventional Radiographs

Conventional radiology (CR) is commonly the first method used to assess back pain. However, radiographs have low sensitivity and specificity, making them less reliable for detecting bone loss. In cases of pyogenic spondylitis, changes may not appear on plain radiographs until 2–8 weeks after the onset of symptoms, and the images may remain normal for several weeks following infection. Detecting bone loss requires a significant reduction in the bone matrix, typically between 30% and 40%, which may take more than two weeks to become evident, especially in acute infections [9,10].

Later manifestations (about 8–12 weeks after) include reactive sclerosis and bony bridge formation between different soma. In the case of effective treatment, the fusion of the vertebral bodies can be appreciated, while in the case of ineffective treatment, a complete somatic collapse can occur [10]. Plain and flexion/extension X-rays should be always performed in a baseline evaluation. However, its specificity in the diagnosis of SI is low (59%) and can at most detect irregularities of vertebral endplates and/or low intervertebral disc height in some advanced cases. In flexion/extension films, a mild instability may be detected.

### 3.2. Computed Tomography (CT) 

CT can detect bone changes earlier than radiography, although it can also be normal within the first three weeks [9,10].

According to the power of CT imaging in evaluating the osseous structures of the spine, it may provide detailed cross-sectional images allowing a precise assessment of bone destruction, and cortical integrity and showing the presence of osteoblastic or osteolytic lesions [12,13].

CT has been demonstrated to have a moderate sensitivity for infections outside the spinal canal but a low sensitivity for spinal epidural abscess [13].

The CT scan can instead be useful in planning and performing biopsies.

CT-guided biopsy is widely used for tissue diagnosis in cases of infective spondylodiscitis, being a safe procedure with a high diagnostic yield and low complication rate; in fact, a biopsy not only helps in confirming the diagnosis but also provides information about drug resistance and bacterial infection [13,14].

### 3.3. Nuclear Medicine

The use of PET/CT imaging for evaluating inflammation and infection is increasingly widespread. Some studies suggest that [18F] FDG PET/CT has comparable diagnostic accuracy to MRI in detecting primary spinal infections, highlighting the complementary role of both methods. In patients with contraindications to MRI or when MRI results are inconclusive, [18F] FDG PET/CT can be a suitable alternative. However, its primary limitation is low specificity, as it cannot distinguish between infection, advanced degenerative disease, or malignancy. Therefore, a comprehensive evaluation of the patient’s medical history is essential [15,16].

If required, 18F-FDG-PET/CT can help to monitor the treatment efficacy [16,17].

Gallium imaging is both sensitive and specific for detecting vertebral osteomyelitis, typically revealing intense uptake in two adjacent vertebrae with loss of the intervening disc. This metabolic imaging technique holds promise in distinguishing between degenerative and infectious diseases. However, radionuclide imaging, including gallium scans, should currently be reserved for cases with uncertain diagnoses or for specific follow-up situations.

Three-phase bone scintigraphy using labeled technetium is a relatively sensitive and specific test; however, it may produce false positives in patients with non-infectious injuries such as fractures and false negatives in early infections or concomitant bone infarction. Labeled leukocyte scans are not useful for the diagnosis of vertebral osteomyelitis. There are also some available PET tracers whose uptake is based on an increased bacterial-specific metabolism. 11CPara-aminobenzoic acid (PABA) is a molecule that has been demonstrated to accumulate in a broad class of bacteria, including Gram-positives, Gram-negatives (as well as *Pseudomonas* spp.), and Mycobacterium tuberculosis. 11C-rifampicin and 2-deoxy-2-[18F] fluoroacetamido-D-glucopyranose ([18F] FAG) have been synthesized and tested in animal settings with promising results [15,16,17].

### 3.4. MR Imaging 

**Figure 1:**
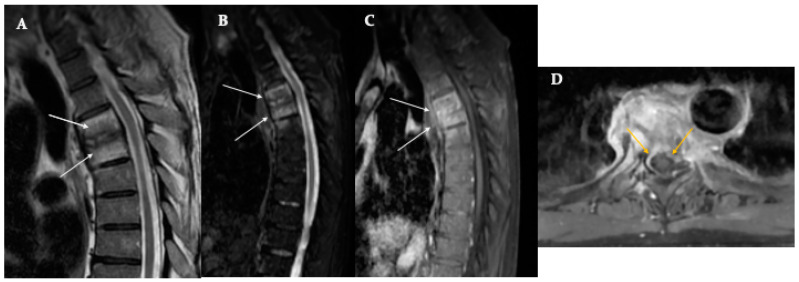
(**A**,**B**) MRI T2-weighted image on the sagittal plane shows marrow edema involving dorsal vertebra reaching up to the endplates (white arrows in **A**,**B**), with great enhancement on post contrast imaging (arrows in **C**) consistent with osteomyelitis. There is an increased amount of fluid signal in the intervertebral disc suggesting a discitis–osteomyelitis complex. (**D**) Post contrast imaging shows enhanced epidural soft tissue (yellow arrows **D**).

MR is the most sensitive imaging technique for the diagnosis of a spinal infectious disease; usually, it is performed with T1- and T2-weighted sequences, on the sagittal and axial planes. The coronal plane can be useful for a more precise evaluation of paravertebral spaces (in the study of the involvement of the psoas muscles, for example). The protocol should always include the fluid-sensitive sequences (such as fat-suppressed (FS) T2-weighted imaging or STIR) because they are highly sensitive in detecting small foci of edema at the initial stage of the disease. The administration of intravenous contrast agents is usually performed on the suspicion of a spinal infection; it improves the visualization of anatomical details and differentiation of phlegmon from epidural abscesses, which is essential because it guides the therapeutic options [18,19,20] (Figure 1).

Epidural abscesses generally require surgical treatment while phlegmon is usually treated with medical treatment. In some cases, when contrast agent cannot be administered, the diffusion weighted image (DWI) technique has been demonstrated to be a valid alternative because of its high sensitivity in detecting spinal and paraspinal abscesses; some authors have demonstrated that these infectious entities show a marked restriction of diffusion on DWI, appearing dark on the ADC map. In addition, DWI can help in differential diagnosis with other spinal diseases, such as hematomas, disc herniation, cystic tumors, facet joint changes, granulation tissue, and fluid collection [21,22,23].

## 4. Imaging Features of Spondylodiscitis of Bacterial Origin

Pyogenic spondylodiscitis usually occurs by hematogenous spread from distant infectious foci in the body. *Staphylococcus aureus* is the most commonly involved organism, accounting for up to >75% of cases.

Patients with Gram-negative infections involving the spine are generally older than those with Gram-positive infections and often have a history of cancer and a recent positive urinary tract infection. Additionally, they exhibit a reduced tendency to develop abscess cavities [24,25,26].

**Figure 2:**
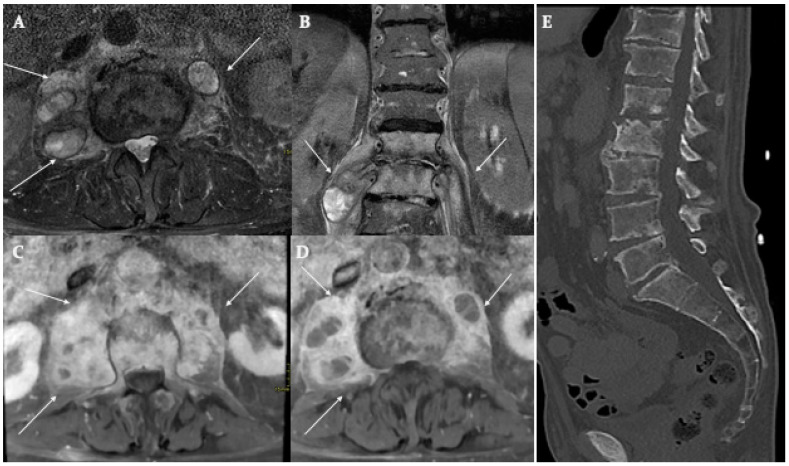
Tubercular spondylodiscitis. Axial (**A**) and coronal STIR (**B**), axial T1 with contrast (**C**,**D**), and sagittal CT (**E**) images demonstrate a severe morpho-structural alteration of the L2 vertebral body and partially of L1, with invasion of the paravertebral space with abscesses affecting the psoas muscles bilaterally (arrows).

**Figure 3:**
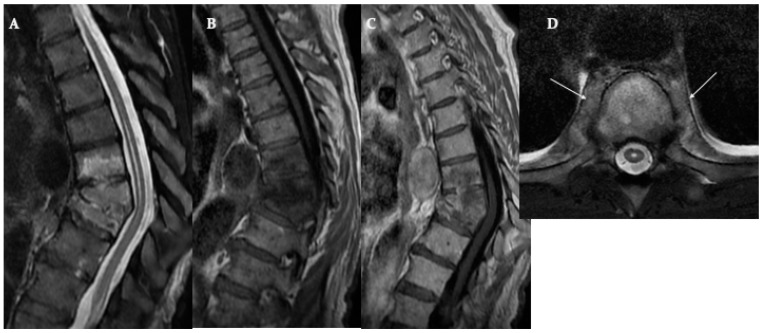
*E. Coli* spondylodiscitis. In the sagittal T2 image (**A**) and T1 image (**B**), osteo-structural alterations with collapse and wedging of the vertebral bodies, accentuation of the physiological dorsal kyphosis, absence of the disc space, and partial vertebral fusion can be observed. In the sagittal (**C**) and axial (**D**) T1 image with contrast, bilateral paravertebral infectious collections are observed (arrows).

In the early stage of the disease, the anterior endplate of the vertebral body is usually affected, showing a high signal intensity on T2W images, which is more conspicuous on FS T2-weighted or STIR sequences; also, the intervertebral disc shows the same changes on T2 images. In most cases, contrast agents may show enhancement of both the bone marrow and the disc. At the very early stage, the disease affects an isolated vertebral body only, and the adjacent disc may be normal. With the progression of the disease, we can observe the erosion of the endplates with the loss of the disc and vertebral height. The paraspinal background also undergoes several changes: from paravertebral edema to the formation of real abscesses or phlegmon within the psoas muscle or diaphragm. In this way, MRI is essential because it is useful and feasible in making the differential diagnosis and guiding the following therapeutic work-up; to obtain the correct diagnosis contrast agent administration is advocated and/or, when this is not possible, DWI can help the diagnosis. DWI may also serve in differentiating postsurgical fluid collections from infected collections because the latter show restricted diffusion due to highly viscous pus (Figure 2 and Figure 3).

In a resolving infection, MRI can show sclerosis and new bone formation with a resolution of the hyperintensity on T2W images [24,25]. In the elderly and immunocompromised patients, the infection may occur at the interfacet joints, showing high signal intensity on T2 images with paraspinal collections in the posterior epidural space.

## 5. Tuberculous Origin (Pott’s Disease)

Spinal involvement in tuberculosis occurs mainly by hematogenous spread.

The thoracolumbar region is the most frequently affected area, with the involvement of multiple vertebrae due to the segmental arterial supply and subligamentous spread of the disease. The infection typically targets the anterior portion of the vertebral body, extending along the anterior longitudinal ligament [27,28,29]. 

**Figure 4:**
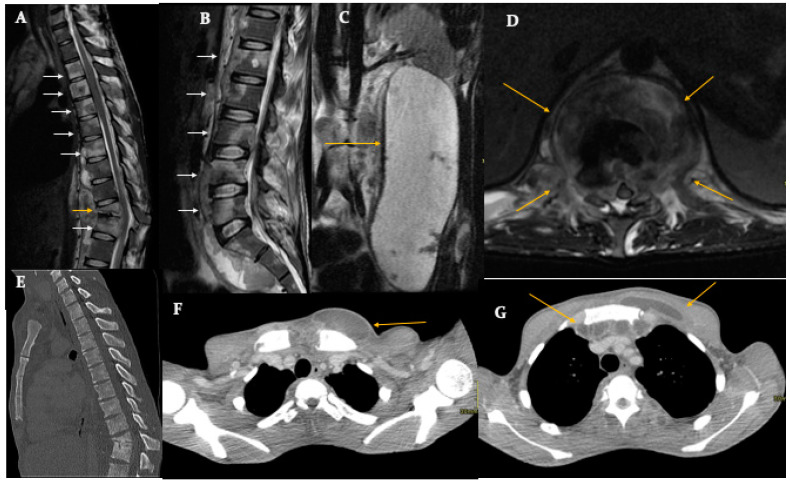
Sagittal fat-suppressed, T2-weighted imaging on the dorsal (**A**) and lumbar (**B**) spine shows multiple vertebral sites of infection (arrows) with signal changes in bone marrow and vertebral collapse (yellow arrow). Coronal (**C**) and axial (**D**) T2-weighted images show a wide collection in the paravertebral space (yellow arrows). A sagittal (**E**) CT scan and axial CT (**F**,**G**) show multiple fluid collection in the parasternal spaces (yellow arrows).

A key imaging characteristic that distinguishes tuberculous infection from bacterial infection is the preservation of the intervertebral disc in the early stages, with disc space narrowing generally being less severe than in pyogenic disease. For this reason, MRI is the preferred diagnostic method for spinal tuberculosis, as it can reveal vertebral body involvement, paraspinal abscesses, vertebral destruction, and spinal deformities, while the disc may not display hyperintensity on T2-weighted images (Figure 2, Figure 3 and Figure 4).

In the chronic stage, hypointensity on T1 and T2-weighted images indicates vertebral collapse with endplate sclerosis, whereas an increase in vertebral signal intensity on T1-weighted images, suggesting fatty replacement, indicates healing. Extension into the spinal canal can lead to arachnoiditis and tuberculomas in some cases when the infection affects the brain. CT is useful to demonstrate bone destruction, endplate erosions, and sclerotic margins, with severe kyphotic angulation in advanced cases (“gibbus deformity”, a deformation observed in TB infections only). The loss of cortical definition and the presence of a calcified and large (larger than that of pyogenic infections) paraspinal mass with irregular rim enhancement are typical of tubercular spondylodiscitis. 

## 6. Brucellar Origin

Spine involvement from Brucella infection represents 6% to 58% of osteoarticular localizations and usually occurs in men over 40 years of age. The lumbar spine is the most commonly affected region (60%), particularly at the L4–L5 level, followed by the thoracic (19%) and cervical spine (12%). Brucellosis may be responsible of a focal or a diffuse form; the latter can be observed in 6% to 14% of cases. From an imaging point of view, brucellar epiphysitis appears as erosions of the superior or inferior vertebral body angle with prominent osteosclerosis, which is a typical presentation of “Pedro Pons’ sign”. The intervertebral disc may be involved either focally or diffusely, with a vacuum degeneration that typically occurs at its anterior aspect, indicating necrosis. Peri-vertebral bone apposition with osteophytosis at the anterior vertebral endplate (parrot’s beak) is typical, occurring earlier than in tuberculous spondylitis. Epidural abscess is a rare complication of spinal brucellosis [30,31,32]. In rare cases, large or calcified paraspinal soft tissue collections may occur, referred to as brucella pseudo-Pott’s disease.

## 7. Post-SARS-CoV-2 Infection

With the widespread pandemic, the literature has reported many cases of spinal related infections after recovering from COVID-19 infection. Among them, many epidural abscesses of various origin have been described, such as fungal and MRSA or tubercular origin. These conditions presented as late complications of COVID-19 infections in patients with multiple comorbidities who have an increased susceptibility to unusual secondary bacterial infections [33,34].

## 8. Mimickers: Differential Diagnosis

### 8.1. Modic Type I Degeneration

When the clinical signs are aspecific and without fever, it can be difficult to determine a differential diagnosis between these two entities.

**Figure 5:**
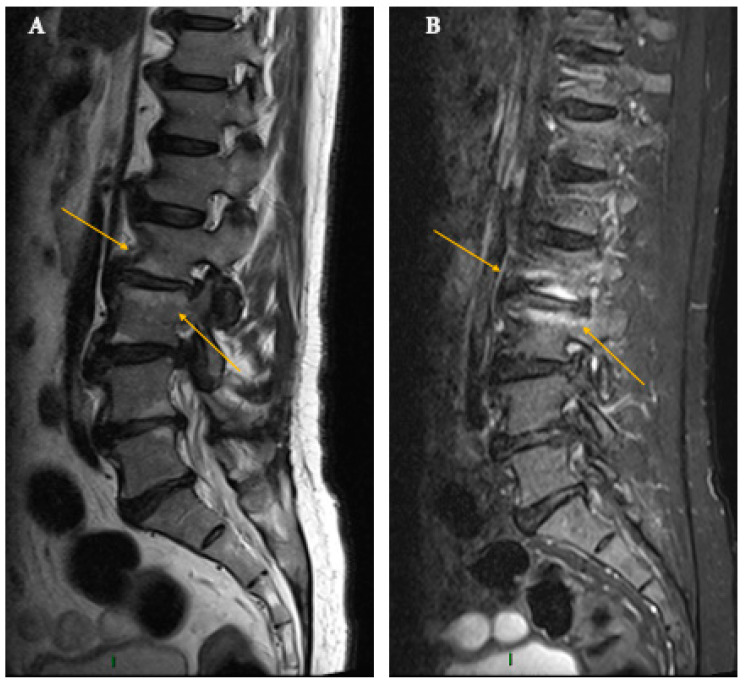
Type I Modic changes at L2–3 endplates showing a hyperintense signal (arrows) on an STIR image (**A**) and a T2-weighted image (**B**). The disc is characterized by degenerative changes, appearing reduced in height. There is no involvement of the surrounding paravertebral edema or psoas muscle.

Modic I alterations typically involve the subchondral bone of two adjacent vertebral bodies associated with degenerative disease of the intervertebral disc; specifically, they appear as areas of high signal intensity on T2- or STIR-weighted sequences and of low signal intensity on T1-weighted sequences, compared to the bone marrow signal. It correlates with pain in some patients. The disc is usually hypointense on T2-weighted images, exhibiting a highly hyperintense appearance of discitis (Figure 5).

DWI may be useful in differential diagnosis since it shows a linear region of restricted diffusion at the interface between normal and diseased marrow within the vertebral body, which is called “the claw sign” [35,36].

Signs in favor of a degenerative change are a lack of paraspinal or epidural collections, lack of disc or endplate enhancement, absence of endplate destructive changes, presence of a degenerative disc space vacuum sign, and its stability over time.

FDG PET/CT has a specificity of 95% in distinguishing Modic type I changes from infectious or inflammatory disease in cases of suspected spondylodiscitis [15].

### 8.2. Acute Schmorl’s Node

**Figure 6:**
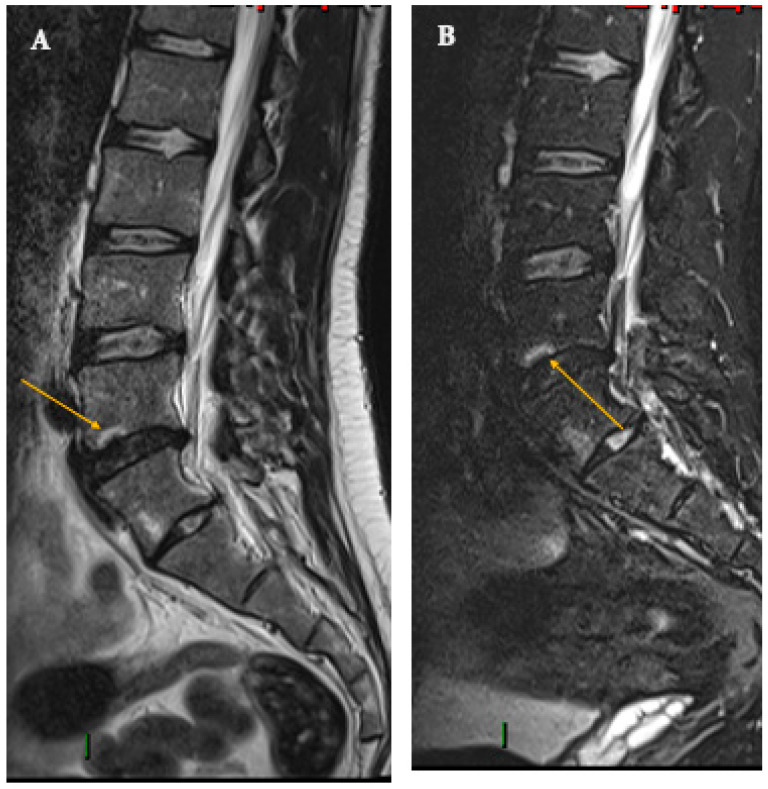
Sagittal T2-weighted image (**A**) and STIR image (**B**) image show a well-defined, crescent nodule (arrow) located in the subchondral area of the L4 body.

This acute condition can mimic early-stage spondylodiscitis but is distinguished by the involvement of only the endplate with a herniated node. It lacks abnormal signal changes within the disc and shows a high-signal-intensity concentric ring surrounding a cartilaginous node (Figure 6). After the injection of contrast media, bone marrow edema and contrast enhancement can be observed because of vascularization and inflammation [37,38].

### 8.3. Ankylosing Spondylitis

Ankylosing spondylitis affects several areas of the spine, mainly at the angular site of the vertebral body (the so-called Romanus lesion), but also at the central level, in the lateral and posterior spinal segments such as the pedicles, costotransverse, costovertebral, and zygapophyseal joints. The natural history of the disease involves erosion changes at the central and peripheral vertebral body, mimicking infectious spondylodiscitis; in these cases, the changes are delimitated by sclerotic borders and usually do not spread outside the vertebral soma with paraspinal collections. MRI is the only imaging technique that can recognize bone marrow edema that, in this kind of disease, is suggestive of active inflammation [39,40]. In the advanced stages of the disease, when disco-vertebral destruction occurs, pseudo-arthrosis develops at the site of injury (the so-called Andersson lesion), appearing in an MRI as endplate erosion and high signal intensity on T2-weighted imaging, as well as the expression of granulation tissue, while the areas of hyperintensity located at the periphery suggest the infiltration of tissue and inflammatory cells, not fluid collection. In these cases, careful evaluation of the ancillary findings, such as the squaring of vertebral bodies, apophyseal joint fusion, syndesmophytes, and a fracture in the spinal posterior elements together with an accurate anamnestic evaluation, is essential for the differential diagnosis.

### 8.4. Osteoporotic Fractures

A vertebral porotic fracture, which appears as the collapse of the endplate, may occur together with paraspinal fluid collection; when these elements are found together, they may mimic infectious spondylodiscitis. In these cases, MRI is essential because it helps to differentiate the two entities, since the vertebral porotic collapse is characterized by a low-signal-intensity rim lining parallel to the endplate, posterior wall retropulsion, and spared normal marrow signal intensity, without disc involvement, which is the most accurate sign of infectious spondylitis [39,41].

### 8.5. Spinal Neuropathic Arthropathy

Patients suffering from neuropathic disorders may develop neuropathic arthropathy which usually occurs in thoracolumbar and lumbar regions. At imaging, this is characterized by vertebral collapse with bone fractures, sclerosis, and new bone apposition and, in the late stage, reduced disc space and pseudoarthrosis. The absence of high signal intensity in T2-weighted images strongly suggests a neuropathic disorder instead of an infectious disease [42,43,44,45].

## 9. Treatment 

The treatment of spondylodiscitis is conservative, based on antibiotics and immobilization of the spine. The antibiotic therapy is based on the causative agent isolated from the cultures, except in cases of neurological impairment or in septic patients. The duration of the therapy is controversial, usually it lasts for 4–12 weeks, and is based mainly on fluoroquinolones, clindamycin, or linezolid; the latter is mainly used in cases of MRSA infection. 

Intramedullary spinal abscess is a rare complication. The overall mortality decreased over the years from 90% to 4%, mainly because of the availability of broad-spectrum antibiotics and new surgical facilities. Early drainage and a rapid administration of intravenous antibiotics are the treatment of choice with a good prognosis; in particular, performing surgical drainage within the first 5 days of symptom onset can result in significantly better neurological outcomes compared to conservative treatment or delayed drainage [46,47,48,49,50].

Spinal epidural abscess is a rare but life-threatening condition with a mortality rate of about 5–16% worldwide. It usually occurs as a complication of primary spondylodiscitis with a hematogenous spread. The most common location is the lumbar spine, followed by the thoracic and cervical tracts. It usually involves more than one segment, which is why a complete study of the whole spine is required [49,50]. Treatment should be started immediately in the presence of a site of infection; in particular, decompression together with systemic antibiotics has been considered the standard of treatment, especially in patients with progressive infection and late diagnosis. Nevertheless, management should be guided by the patient’s parameters, as good outcomes have also been demonstrated for conservative treatment. In fact, conservative management should be performed in patients with a wide spinal involvement, in cases with an early diagnosis and with minor neurological involvement, in those with severe comorbidities who cannot undergo surgery, and in patients presenting with complete paralysis for more than 72 h. Particular attention should be paid to patients suffering from diabetes mellitus, neurological disease with spinal involvement, Methicillin-resistant *Staphylococcus aureus* (MRSA) infection, alterations of lab tests (CRP and WBC), bacteremia, and ring-like enhancement on MRI [47,48]. The surgical options consist of minimally invasive or endoscopic procedures, segmental decompression, and ventral debridement of the disc. The choice of surgical approach primarily depends on the nature of the lesion—whether it is solid (granulation tissue) or liquid (abscess). Patients who undergo surgical treatment have shown favorable outcomes in over 60% of cases. These results have led some authors to advocate for surgical intervention even in the absence of neurological deficits, particularly when lesions are situated in the cervical or thoracic spine [47].

Subdural empyema typically arises from a hematogenous infection or the spread of infection from osteomyelitis. MRI with gadolinium is the preferred diagnostic method, followed by CT with myelography. Treatment usually involves surgical drainage, followed by targeted antibiotic therapy. Depending on the extent of the lesion, a (hemi-)laminectomy over one or more levels may be needed. In cases of more extensive spread, flavectomy or laminectomy at multiple levels may be necessary to fully remove the infectious material [48,49,50].

### Paraspinal and Iliopsoas Abscess

The treatment of a paraspinal abscess is usually the same as the primary spinal infection, requiring surgical drainage when a debridement is performed for the spondylodiscitis or an antibiotic treatment when a non-surgical approach is the option [51,52,53,54,55].

Iliopsoas abscess is a common complication of an inflammatory spinal disease and it usually requires broad spectrum therapy, covering *Staphylococcus aureus* as the most common pathogen involved. When the conservative management fails, the treatment of choice demanded is image-guided drainage, which is demonstrated to be a safer option than open surgical treatment. The latter is indicated in the presence of a multiloculated abscess with a wide spinal infection.

## 10. Discussion

Spinal infections are a rare entity with an increasing incidence. If not early discovered, they can be potentially fatal. MRI is the preferred imaging technique for diagnosing spinal infections, showing some specific features that can help to recognize a bacterial or non-bacterial infection, as well as complications, such as epidural abscess or muscle abscess. In addition, it may help to guide the treatment, suggesting a conservative or surgical approach depending on the disease extent and the involvement of paraspinal structures. The MRI protocol should always include fluid-sensitive sequences (such as fat-suppressed (FS) T2-weighted imaging or STIR) because they are highly sensitive in detecting small foci of edema at the initial stage of the disease. In a typical infection, the disease first involves the anterior aspect of the vertebral body in the metaphyseal region and then reaches the intervertebral disc and the adjacent vertebral body. Vertebral involvement appears as a T1 hypointensity and a T2 hyperintensity with contrast enhancement, because of the presence of marrow edema, and is often more pronounced along the endplates at the infected level. Some studies have shown that T1 hypointensity and contrast enhancement are more frequently observed than T2 hyperintensity in the bone marrow [20,21,22,23,24]. In the late stage, it is common to observe the endplate erosions and vertebral body destruction. Disc involvement is characterized by T2 hyperintensity, disc space height loss of the intervertebral disc, loss of the normal T2 hypointense intranuclear cleft, and contrast enhancement. Disc enhancement pattern is variable. The administration of intravenous contrast agent is usually performed because it improves the visualization of anatomical details and differentiation of phlegmon from epidural abscesses, which is essential because it guides the therapeutic options. When contrast agent cannot be administered, DWI can be a valid alternative because of its high sensitivity in detecting spinal and paraspinal abscesses; some authors have demonstrated that these infectious entities show a marked restriction of diffusion on DWI, appearing dark on the ADC map. In addition, DWI can help in the differential diagnosis of other spinal diseases, such as hematomas, disc herniation, cystic tumors, facet joint changes, granulation tissue, and fluid collection. MRI guides in the differential diagnosis of other spinal diseases which can mimic a spinal infection and, along with clinical parameters, helps to obtain the correct diagnosis. The findings considered highly sensitive for pyogenic spondylodiscitis include paraspinal or epidural inflammation, vertebral body T1 hypointensity, disc space T2 hyperintensity, and disc space enhancement. On the other hand, findings with relatively low sensitivity include disc T1 hypointensity and height loss. While MRI is highly effective for diagnosing spinal infections, it becomes more challenging in cases of postoperative infection. Discitis and osteomyelitis are rare complications after lumbar spine surgery, and in such cases, MRI is less reliable, as normal postoperative changes can still show signal alterations in the disc or endplate. In fact, MRI cannot differentiate between pathology and normal postoperative alterations until at least 6 months after surgery. A key indicator of surgical changes rather than infection is the presence of two parallel thin bands of enhancement in the disc space, in contrast to the diffuse, amorphous enhancement typically associated with infection [23]. Paravertebral enhancement supports the diagnosis of infection, while the absence of enhancing, edema changes, or disc space enhancement makes infection less likely. CT is particularly useful where MRI is contraindicated, not available, or equivocal. It is useful to confirm the suspicion of degenerative disc changes in patients who have been referred for biopsy, such as the disc space vacuum phenomenon, which may obviate the need for biopsy. Nuclear medicine imaging is used only in specific situations, because of its limited spatial resolution, long examination time, and low availability, in contrast to the high spatial resolution and excellent diagnostic performance of MRI.

Differentiating pyogenic spondylodiscitis from other conditions, such as Modic type 1 changes, can be challenging. MRI plays a vital role in diagnosing both spinal epidural and subdural abscesses, which typically require surgical treatment. Tuberculous spondylitis, the most common granulomatous spine infection globally, is characterized by features like disc space preservation, multilevel involvement, and large abscess formation, which strongly suggest the diagnosis. Brucellar spondylitis is more prevalent in endemic areas and, while it can resemble tuberculous spondylitis, it usually presents with a less aggressive appearance.

## 11. Conclusions

Spondylodiscitis is an infective disease with a bacterial or non-bacterial origin. In the early phase, it may have an insidious onset with no specific clinical features; for this reason, imaging plays a pivotal role in the recognition and diagnosis of the disease, helping in differential diagnosis with other entities displaying the same imaging and biological behavior. This information is crucial in order to determine the correct treatment as soon as possible, as spondylodiscitis is considered a life-threatening condition, which could lead to dangerous complications requiring, in such cases, a surgical approach.

## Data Availability

The original contributions presented in the study are included in the article; further inquiries can be directed to the corresponding author/s.
